# Comparison of the superimposition accuracy in the integration of different digital dental models into the cone-beam computed tomography: An ex vivo study

**DOI:** 10.4317/medoral.27724

**Published:** 2025-11-22

**Authors:** Daniel Amaral Alves Marlière, Maria Júlia Assis Vicentin Calori, Luciana Asprino

**Affiliations:** 1 Department of Clinical Dentistry, Dentistry School, Federal University of Juiz de Fora, Juiz de Fora, Minas Gerais, Brazil; 2 Division of Oral and Maxillofacial Surgery, Piracicaba Dental School, State University of Campinas, Piracicaba, São Paulo, Brazil

## Abstract

**Background:**

Accurate virtual planning for orthognathic surgery and dental implants requires integrating cone-beam computed tomography (CBCT) images into digital dental models, but the impact of different scanning methods on the superimposition accuracy remains unclear. The aim of this study was to compare the superimposition accuracy of dental models obtained from two scanning methods using CBCT scans of dental surfaces.

**Material and Methods:**

The maxilla (MX) and mandible (MD) of 4 dry skulls were scanned using CBCT and converted into 3D meshes. Dental arches were obtained using intraoral devices (IS) and by digitizing plaster models using an extraoral scanner (PM). Sixty-four digital models were produced per group. Each 3D mesh and corresponding model were imported into the Geomagic software for alignment and deviation analysis. Registration errors and mean deviations (MD+ and MD-) were assessed quantitatively, and the qualitative evaluation was performed through color maps.

**Results:**

Deviations ranged from -0.4 to 0.3mm in both groups, with 80% of the values distributed between -0.5 and 0.5mm. In both groups, Registration error, MD+ and 3D Error were below 0.5mm, and MD- was higher than -0.5mm. Statistically significant differences were found between IS and PM models for MD+, as well as for registration error and MD+ in mandibular comparisons.

**Conclusions:**

Superimpositions were not affected by the model acquisition method, and both were clinically acceptable. However, PM superimpositions showed greater deviation magnitudes, suggesting an inclination for IS to produce more accurate alignments, especially in the mandible.

## Introduction

The virtual simulation and planning transfer workflows in orthognathic surgeries, as well as the placement of dental implants, were developed using computer-aided design and manufacturing (CAD/CAM) technologies. The exclusive use of cone-beam computed-tomography (CBCT) images does not yield dental surface details due to limited resolution caused by artifacts resulting from the presence of metallic restorations, orthodontic devices, as well as dental-enamel mineral density (1 - 3). For virtual planning of precise bony and dental structures, CBCT acquisitions provide digital maxilomandibular bone-tissue and dental-arch model images to replace the dental surfaces shown in the CBCT (4). To overcome the limitation of the CBCT images, there are a number of studies presenting registration, superimposition or fusion techniques to integrate digital dental-arch models into the dental surfaces using CBCT images (2 , 4 - 12), which were replaced to optimize the image quality, minimizing tooth magnification and allowing for the presence of surfaces closer to the 'natural' state of the 3D reconstructions (8 , 13). The iterative closest point (ICP) algorithm was used as the operational basis to integrate the virtual dental models into the tomographic images considering state of the art registration techniques (14 , 15), regardless if the methods applied used fiducial markers or not, if the alignment of the CBCT images and virtual dental models was based on a combination of referenced anatomical points or superimpositions due to the similarity of the surfaces (16), or if there was the presence of a higher number of artifacts in the CBCT images (17). By quantifying the error magnitude in the processing of these file registrations, it is possible to determine the accuracy of integrating these digital files, which may impact the clinical results (such as orthognathic surgeries or dental implants). The 0.5-mm error magnitude was deemed acceptable for clinical applicability without interfering with the orthognathic surgery or dental implant outcomes (5 , 8). Nonetheless, no previous studies have compared superimpositions of different model types (plaster models scanned using a desktop scanner or intraoral scanner) over 3D meshes resulting from the CBCT. Within this context, it is questioned whether the superimposition accuracy could interfere with the viability when integrating and replacing dental surfaces of CBCT images with different digital dental models. This study aimed to assess the superimposition accuracy of digital dental models derived from different dental image scanning methods over CBCT image dental surfaces. It compared the registrations obtained with plaster models using the extraoral scanner with those obtained using the intraoral scanner on dental surfaces.

## Material and Methods

This ex vivo experimental study (using dry skulls) was developed at the Piracicaba Dentistry School (Campinas, Piracicaba, and São Paulo State Universities) Oral Diagnosis Department and approved by the Research Ethics Committee of the University of Campinas' Piracicaba Dentistry School under number 35150920.3.0000.5418 (Certificate of Presentation for Ethical Consideration). Setting and sample Between February and April 2021, 320 dry human skulls were analyzed. The inclusion criteria were: Presence of the maxilla together with the respective mandible from the same dry skull containing intact bony structures in the dental-alveolar regions and absence of anatomical abnormalities; partially intact permanent dentition from the right third or second molar to the left third and/or second molar in the maxilla and mandible; and the presence of at least 24 teeth (18 , 19), reaching a total of 316 excluded skulls. The four selected skulls were identified by number and associated with their corresponding maxillae (MX) and mandibles (MD). After being identified, each skull MX and MD was sequentially submitted to a workflow that began with CBCT image acquisitions, followed by the production of plaster models that were later digitized using a desktop scanner (extraoral scanner), and dental surface scans obtained with an intraoral scanner. According to the numerical variables associated with a 95% test power and a 5% significance level, the calculation defined a minimum sample size of 16 digital dental models, both in MX and MD dental arches, to be compared with the 3D meshes resulting from the dry-skull CBCT images. CBCT image acquisition and processing Each dry skull was submitted to image acquisitions by means of CBCT scanning using the OP 300 Maxio (Instrumentarium Dental, Tuusula, Finland) device with a 13x154-cm FOV, 0.4 mm/voxel resolution, X-ray source parameters at 90KVp tension, 8.0 mA current, 360° rotation, and 10-second acquisition times. Each MX and MD skull was separately adapted in polypropylene flasks filled with water to reduce radiation (20 , 21). The MX and MD were sequentially submitted to CBCT image acquisitions by the same radiology technician. Eight file volumes in the Digital Imaging and Communication in Medicine (DICOM) format were generated, and each skull was identified as MX or MD (1, 2, 3 and 4). Based on the DICOM files, an operator (LFB) imported each file volume into the Materialise Mimics software, version 23.0 (Materialise NV, Lovaine, Belgium), to convert each DICOM file into a 3D mesh. The segmentation was performed manually in the software, stipulating a minimum (226 HU) and maximum (2619 HU) gray-scale range limit. Regions with image artifacts were removed manually. The software selected optimized quality (calculate part - optimal) to produce the 3D meshes, which were converted into Standard Tessellation Language (STL) file formats. Dental arch scan (intraoral scanner) The dry skull MX and MD dental arches were scanned using the CS3600 Access (Carestream Dental, Atlanta, Ga) intraoral scanner coupled to a portable computer (Avell High Performance, Manaus, Amazonas, Brazil). The scanner digitization tip was calibrated for 2 minutes before starting the scan. The dental surface scans followed all parameters recommended by the manufacturer and the technique described by Sun et al. (22). The scans were performed 8 times for each maxillary and mandibular dental arch by a single operator (DAAM) within a 5-day period. The digitized dental arches were filed in the STL format and identified according to the MX and MD dental arches, followed by the numerical identification of each skull and the IS initials referring to the dental-model group resulting from the intraoral scan, in a total of 64 3D meshes (32 MX-IS and 32 MD-IS). To define the region of interest and delete outlier regions, the operator (LFB) removed the region adjacent to the dental crowns to standardize the number of triangles/polygons on the model surfaces, reducing the possibility of result bias. Plaster model manufacture and scan Two operators (DAAM and MJAVC) modeled each MX and MD dry-skull dental arch eight times over five days using Type I Hydrogum 5 irreversible hydrocolloid (alginate) (Zhermack Dental, Badia Polesine, Italy), adhering to the dry skull sequence. Next, the molds were filled with special Durone type-IV plaster (Dentsply Sirona, São Paulo, Brazil). The plaster models were identified according to the respective MX and MD dental arches, followed by each dry skull and PM-group numerical identification. Transversal linear measurements were taken on the type of canine cusps (13 up to 23 and 33 up to 43), second premolar vestibular cusps (15 up to 25 and 35 up to 45), and second-molar mesio-vestibular cusps (17 up to 27 and 37 up to 47) in all PM-group models. The measurements were independently performed twice by each evaluator (DAAM and MJAVC) within a 30-day period. These plaster models were stored at room temperature in a dry environment and digitized using a Ceramill Map400 (Amann Girrbach, Koblach, Austria) extraoral scanner. All files were set to the STL format, in a total of 64 3D meshes (32 MX-PM and 32 MD-PM), which were also standardized by removing the region adjacent to the dental crowns, as previously mentioned (LFB operator). 3D registers and analyses Each MX or MD STL model resulting from the CBCT was simultaneously imported with the respective STL MX or MD dental model file resulting from the IS or PM group into the reverse engineering computing program Geomagic Qualify 2012 (3D System, California, USA). In this computing program workflow, the operator (LFB) applied the methodology described in the study by Marlière et al. (23) or a similar approach. Each MX or MD resulting from the CBCT was defined as a Reference to be superimposed over each Test 3D-mesh dental model from each IS or PM group. In pairs, matching each CBCT 3D mesh with the respective dental model, the program applied the ICP to register and analyze the deviations (accuracy assessment) between the 3D meshes. From the categorization to the Reference 3D meshes with the respective Test model in the computing program, the object-moving tool enabled the approximation of each Test mesh to its respective Reference, manually overimposing the surfaces by similarity. Then, the program provided semi-automatic superimpositions of each 3D mesh using the Best-fit Alignment tool. Based on the model superimpositions, the program generated the Model Maximum Length and Average Error values. The 3D Compare tool was used for each Reference or Test-mesh pair, calculating the Euclidean distances in a minimum scale of -1mm to 1mm. This tool provided the positive mean deviation (MD+) and negative mean deviation (MD-) values, reflecting the positive and negative deviations between the overimposed points of the respective 3D meshes, which may cancel each other out in the absolute mean deviation calculation. This tool also generated the root mean square (RMS) values for all point-pair distances between the meshes, regardless of whether each deviation value was positive or negative, by squaring each deviation value, summing the squares, and then taking the square root to estimate the RMS value (23). The RMS value was considered the 3D Error. These variables were registered in millimeters (mm). This same software function also produced qualitative results, showing color dispersion on the dental surfaces and comparing the 3D congruency between the Reference and Test meshes. The 3D comparison of the Reference and Test meshes supplied a percentage deviation (%) analysis between the point correspondences for each 3D-mesh pair, which quantified and categorized the distribution of deviation values according to the deviation-value intervals within, or not, the clinically acceptable value range: % clinically acceptable deviation (-0.5 Deviation 0.5); and % clinically unacceptable deviation (-1&lt; Deviation &lt;-0.5 and 0.5&lt; Deviation &lt;1). The same operator (LFB) measured the deviation vectors between the Reference and Test meshes through 6 points distributed on the dental surface of the overimposed pairs in the following regions: Incisal medial point of the central incisors (11 and 21; 31 and 41); canine cusps (13 and 23; 33 and 43); and second molar mesio-vestibular cusps (17 and 27) and disto-vestibular cusps (37 and 47). For each point determined by the operator, the tool supplied the measured 3D-Deviation vector, along with the x, y, and z vector measurements. Consequently, the 3D Deviation value was obtained for each point indicated on the dental surfaces in the overimposed 3D-mesh pairs. The stratified values for vectors x (mediolateral deviation), y (anteroposterior deviation), and z (superoinferior deviation) were named Dx, Dy, and Dz, respectively. The variables were registered in millimeters (mm). According to previous studies (5 , 8 , 24), a reference (e.g., a measurement criterion) was established at ±0.5mm to assess the acceptability and applicability of the registers, without interfering with the workflow for virtual planning of orthognathic surgeries and dental implants. Statistical analysis To verify the reproducibility and reliability of the PM models, the coefficient measurements obtained on the MX and MD cusps were subjected to descriptive analyses and the Pearson correlation test (r) to assess intra- and inter-evaluator agreement. To assess each replica (independent observation) of the experimental unit (dry skull), results from deviation analysis (Registration Error, MD+, MD-, and 3D Error) were submitted to Welch t and U-statistic (Wilcoxon-Mann-Whitney) tests according to the sample distribution and associated with the size of the computing effect using Cohen's d correlation and rank biserial correlation (r), respectively. The values from the alignment and deviation analysis were submitted to descriptive statistical analysis. The variables were tested for normality of distribution using the Shapiro-Wilk test (p&gt;0.05). The Wilcoxon test was applied to check if the null hypotheses of the values obtained in Registration Error, MD+, and 3D Error would be smaller than or equal to 0.5mm (H0: µ0.5); or for MD-, greater than or equal to -0.5mm (H0: µ-0.5) (8 , 24). The Kruskal-Wallis and Mann-Whitney tests were used to detect if there was a variable-distribution difference between the PM and IS groups (Registration Error, MD+, MD-, and 3D Error), and to check the null hypotheses if the PM group results were statistically lower than or equal to the distribution for IS (H0: µPM µIS); and, higher than or equal to the distribution for IS, specifically for the MD- (H0: µPM µIS) variable. These tests were also used to assess whether there would be differences in the percentage quantification of the deviations distributed across the 3D-mesh surfaces. The results obtained for the points on the occlusal surfaces (3D, Dx, Dy, and Dz deviations) could yield positive or negative values and were submitted to modules to confirm the positive values. The Kruskal-Wallis and Mann-Whitney tests were applied to compare the superimposition deviations between PM and IS (H0: µPMµIS); and between the anterior-teeth deviations (11, 21, 31 and 41) in comparison with the posterior teeth (17, 27, 37 and 47) in each PM and IS group (H0: µAnteriores µPosteriores). The statistical analyses were performed using the R Core Team computer program, version 4.1.2 (R Foundation for Statistical Computing, Vienna, Austria), with a statistical significance level of p0.05.

## Results

**Figure 1:**
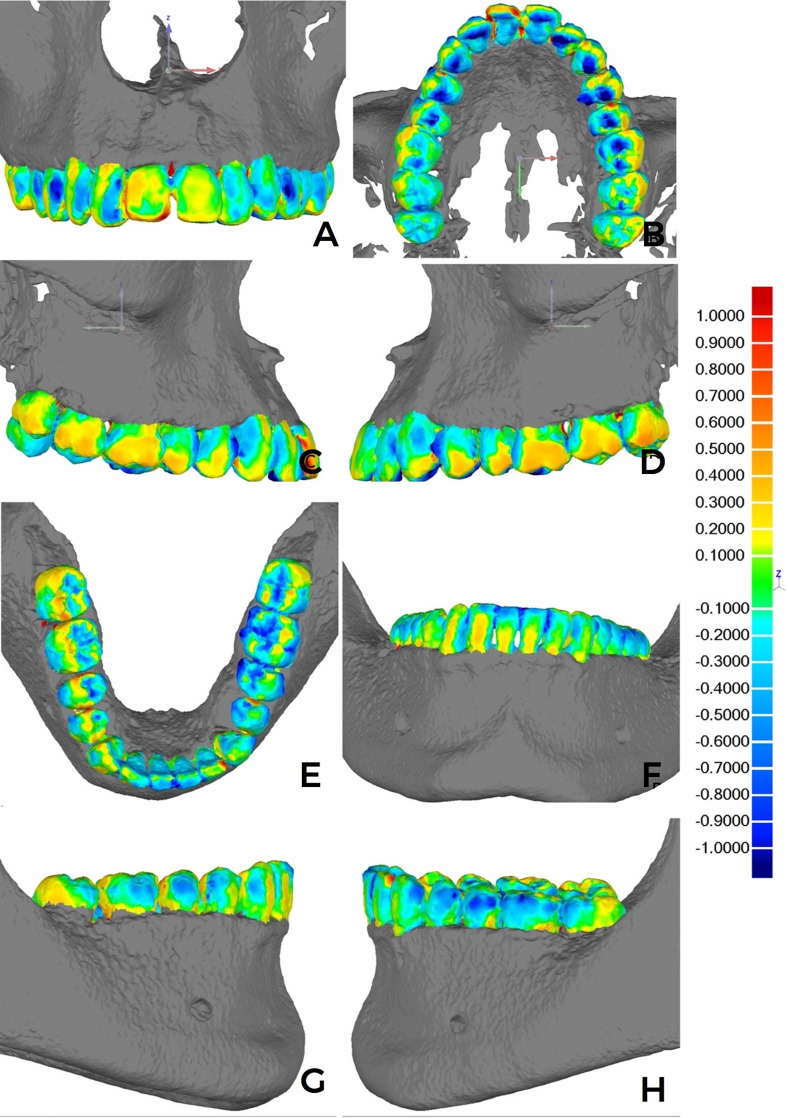
Color map images after the registration and analysis of deviations in millimeters (mm) on Geomagic. (A-D) MX-IS: (A) frontal view of the anterior and posterior-tooth crown vestibular slopes, (B) occlusion view, and (C-D) lateral view. (E-H) MD-IS: (E) frontal view of the anterior and posterior-tooth crown vestibular surfaces, (F) occlusion view, and (G-H) lateral view.

**Figure 2:**
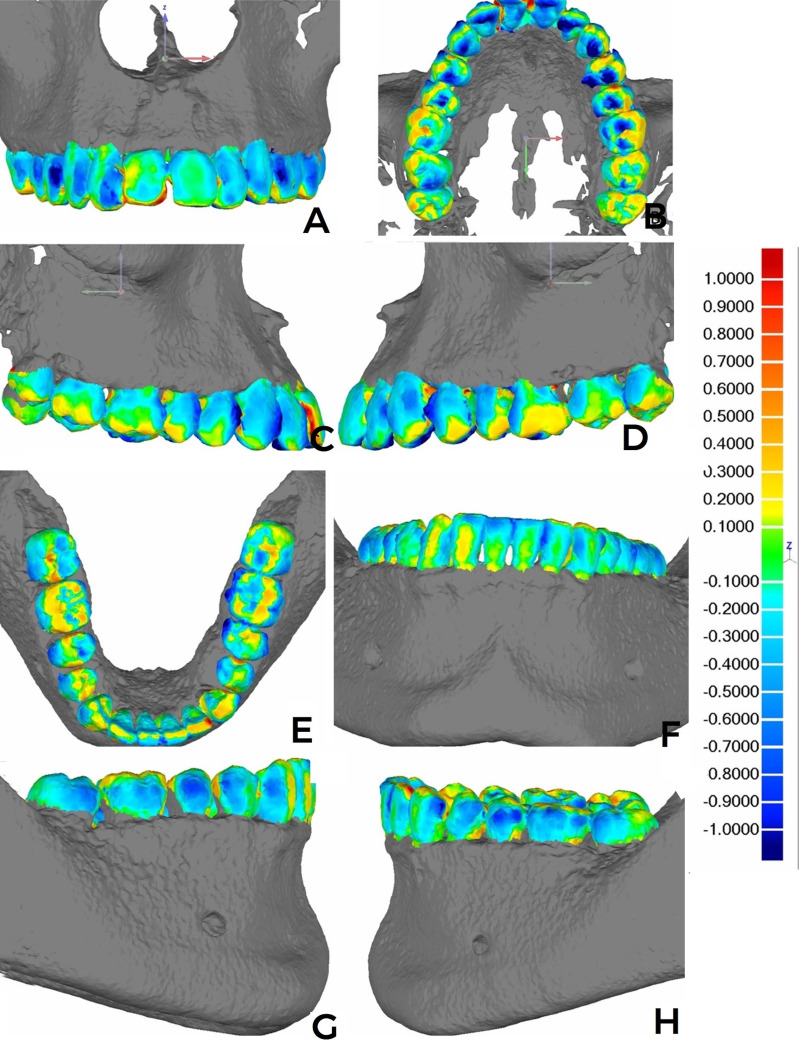
Color map images after the registration and analysis of deviations in millimeters (mm) using Geomagic. (A-D) MX-PM: (A) frontal view of the anterior and posterior-tooth crown vestibular slopes, (B) occlusion view, and (C-D) lateral view. (E-H) MD-PM: (E) frontal view of the anterior and posterior-tooth crown vestibular surfaces, (F) occlusion view, and (G-H) lateral view.

Intraevaluator coefficients varied between 0.85 and 0.99, whilst the interevaluator coefficients varied between 0.84 and 0.99, indicating excellent PM group-model reproducibility (Supplementary File 1. http://www.medicina.oral.com/carpeta/suppl1_7724). Regarding the replicability of the registration process, the Model Maximum Length absolute values were identical for every registration, considering the specific dental arch and type of scanning (Supplementary File 2. http://www.medicina.oral.com/carpeta/suppl2_27724). The color map for each registration combination indicated mesh deviations ranging from -0.4mm to 0.3mm. The dark blue and red tones were less pronounced in the surface dispersions. Both for maxillary and mandibular arches, the presence of colder tones was observed in occlusal sulci and/or fissures, and hotter tones in interproximal and cervical points of the dental crowns close to the alveolar-rim region (Figures 1 and 2).

1

2

There were no striking and visibly discrepant differences in the color dispersion between the IS and PM groups. Whenever noticeable, there were discrete differences of a greater preponderance of light and dark-blue tones on the vestibular surfaces of the teeth (premolars and molars) for the MX- (Figure 2 A-D) and MD-arch (Figure 2 E-G) PM model superimpositions. Over 80% of the deviation dispersion between the overimposed meshes was concentrated within the -0.5mm and 0.5mm deviation-value range. There were no statistical differences between IS and PM, considering associated or independent arches according to MX and MD (Table 1).

Table 1

The mean, median, minimum, and maximum values of the descriptive results for the Registration Error, MD+, MD-, and 3D Error variables are included in Table 2, considering both IS and PM groups, along with the associated or independent MX and MD dental-arch superimpositions.

Table 2

**Figure 3:**
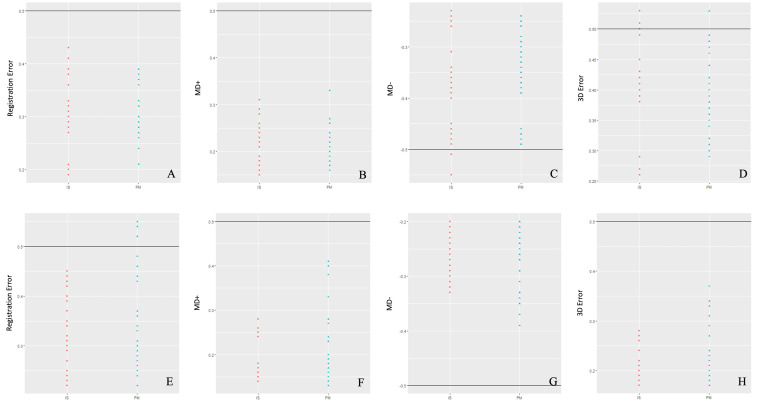
Bland-Altman plots show the dispersion of the alignment-error variables and analyses of the deviation superimpositions for the IS and PM groups, with (A-D) MX dental arches and (E-H) MD dental arches.

The Shapiro-Wilk test results indicated that these variables did not present a normal distribution (p&lt;0.05). The Wilcoxon test results did not reject the null hypothesis (H0: 0.5) of the variable values lower than or equal to 0.5mm (p&gt;0.05) (Figure 3).

3

**Figure 4:**
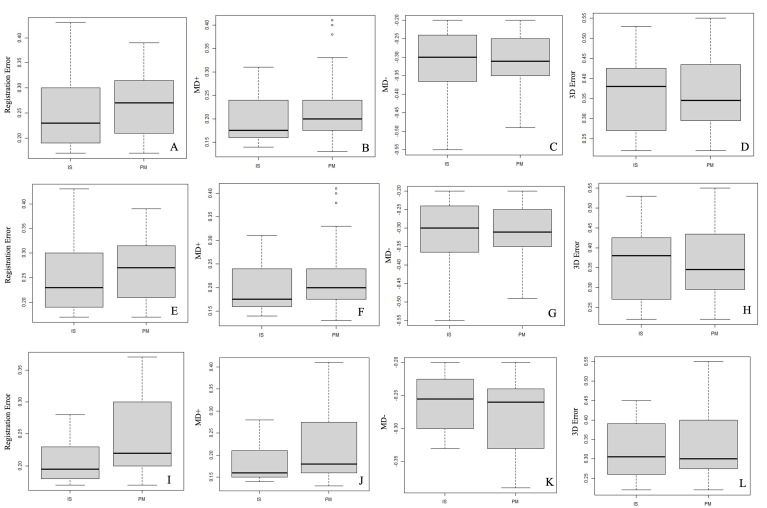
Box-plot graphs present the distribution of the alignment-error response variables and an analysis of the deviations comparing the IS and PM group superimpositions, with (A-D) associated dental arches; (E-H) MX dental arches; and (I-L) MD dental arches.

When considering the comparison between the IS and PM group superimpositions (matched dental arches), there was no statistical difference in the distribution of the variables for Registration Error (p=0.07), MD- (p=0.59), and 3D Error (p=0.74) (Figure 4A, C, and D). According to the test results, a statistically significant difference was observed for the MD+ (p=0.01) distribution when comparing the IS and PM, which rejected the null hypothesis (H0: µPMµIS) (Figure 4B). To compare the distribution differences for each variable according to the IS and PM groups on a specific dental arch, the Kruskal-Wallis test results did not indicate a statistical difference between the variable answers for Registration Error (p=0.76), MD+ (p=0.43), MD- (p=0.48) and 3D Error (p=0.37), specifically considering MX arches (Figure 4E-H). To compare the superimpositions between the IS and PM groups in MD arches, the results did not determine statistical differences between the MD- (p=0.28) and 3D Error (p=0.43) variables (Figure 4K and L), with statistical differences being observed in the Registration Error (p=0.01) and MD+ (p&lt;0.01) (Figure 4I and J) results, indicating that the distribution for these two variables was statistically higher in the PM group superimpositions, rejecting the null hypothesis (H0: µPM µIS).

4

The mean and median values of the points determined on the teeth were not higher than or equal to 0.5mm for the IS and PM group superimpositions. Most of the results (3D, Dx, Dy, and Dz deviations) did not show statistically significant differences between the IS and PM groups in either dental arch. For the comparisons between the maxillary IS and PM superimpositions, there were statistical differences (p0.01) for 3D Deviation, Dy, and Dz (tooth 21); Dx (tooth 23); Dx (tooth 13); and Dy (tooth 27). In the mandibular arch IS and PM superimpositions, significant statistical differences (p0.02) were observed in 9 points: Dx deviations (tooth 31); 3D deviations, Dx, Dy, and Dz deviations (tooth 33), 3D and Dz deviations (tooth 37); and 3D and Dz deviations (tooth 41) (Supplementary File 3. http://www.medicina.oral.com/carpeta/suppl3_27724). When analyzing the results of the 3D and vectoral deviations (Dx, Dy, and Dz) for anterior teeth compared to posterior teeth, no statistical difference was observed considering the right maxillary arch teeth in both groups. On the left maxillary arch, there was a significant difference for the Dy vectoral deviation in the IS group, and 3D and vectoral (Dx, Dy, and Dz) deviations in the PM group. For the mandibular arch in both groups, statistical differences were observed in the 3D and vectoral deviations (p&lt;0.05) (Supplementary File 3 http://www.medicina.oral.com/carpeta/suppl3_27724).

## Discussion

This study employed an experimental research method using dry skulls and a standardized laboratory methodology, from acquisition to image processing, to minimize potential result biases. In addition, 3D meshes from digital dental models in both groups were standardized (the adjacent area next to the dental surface was removed) to achieve a better fit in the region of interest, thereby avoiding outlier rejection and facilitating convergence tolerances according to the established threshold. Even if all results from deviations were grouped in IS and PM categories, we might have hypothesized that each digital dental model (replica) would be considered a pseudo-replica (identical or not, depending on the independent observation), since they were manufactured sequentially by four experimental units. However, we conducted an additional statistical analysis, which indicated that the replicas (dental models) would not influence our results, as most p-values were lower than 0.05, with medium to large effect sizes in most of the results (Supplementary File 4). http://www.medicina.oral.com/carpeta/suppl4_27724. Firstly, CBCT is not precise in reproducing dental structures, as metal restorations, orthodontic devices, dental implants, and variations in tooth enamel density can introduce artifacts that affect the beam-hardening effect, thereby compromising tomographic image resolution (4). To minimize the deterioration of dental images in 3D CBCT reconstructions, Kang et al. (25) have applied gray-value thresholds during the segmentation of tomographic images, before the fusion of digital dental models. According to their method, the authors specified a gray value range during image segmentation, varying from a minimum of 226 HU to a maximum of 3017 HU. To perform the registration between CBCT images and 3D meshes from intraoral scanning for guided surgery in dental implants, Flügge et al. (5) carried out two types of 3D tomographic reconstructions for bone with a stipulated limit ranging from a minimum of 350 HU up to a maximum of 2250 HU or manually determined by the operator (without a standardized gray value). From their results, segmentations standardized by a computer program affected the inaccuracy of tooth surfaces in CBCT 3D meshes, which also resulted in higher inaccuracy in the overlays with digital dental models. To minimize these effects in this study, CBCT image processing focused on establishing a uniform standard for the CBCT 3D meshes, manually matching the bone segmentation, and stipulating a gray value variation limit between a minimum of 226 HU and a maximum of 2619 HU. It was carried out to optimize the 3D mesh surfaces and maintain the most anatomically accurate morphology of the teeth, facilitating the superimposition process. However, anatomical structures in CBCT may still be compromised when the segmentation limit range is standardized due to the inherent lack of gray-value homogeneity in HU units (10). Many studies have utilized fiducial markers to register digital dental models between 3D meshes (6 , 11 , 12). Nevertheless, we opted not to use markers, as we agree with previous studies that indicate it is unfavorable for clinical applicability in orthognathic surgery (6 , 11 , 12). Moreover, the integration of digitized models with CBCT images was viable, eliminating the need for adding markers, thereby achieving higher accuracy when using the surface-based method instead of the point-based method (16). In this study, there was no need for adapted markers on dry skulls reproduced on physical and digital dental models, nor the indication of anatomical points on the model surfaces, as the methodological process employed a surface-based method to superimpose the 3D meshes. In addition, the absence of fiducial markers did not influence the registration process, as the Model Maximum Length results were similar to each other (Supplementary File 2. http://www.medicina.oral.com/carpeta/suppl2_27724). This allowed for the estimation of an absolute number of iteration processes, connecting consistency to the reproducibility of the registers. In our study, we did not evaluate the intra- and inter-operator reproducibility of the workflow process in Geomagic software, as repeating the entire process with STL files replicated in the same software would be irrelevant, given that it has already been tested (13 , 23). Furthermore, it would be reasonable to recognize that STLs were replicated 8 times for either the maxilla or mandible, and the values related to the Maximum Model Length represented numbers of interactions of all points dispersed between each pair of STL files, which were identical (Supplementary File 2. http://www.medicina.oral.com/carpeta/suppl2_27724). In particular, the plaster dental model workflows depended on a hand-manufacturing process using dental materials, which could provide distortions despite all care taken in the methods. Thus, it is more critical to obtain appropriate reproducibility to prove that the plaster dental models manufactured were reliable (Supplementary File 1. http://www.medicina.oral.com/carpeta/suppl1_27724), minimizing possible bias in comparisons between PM and IS. Although dental models from the IS group had not been measured before registration, we based our results on previous studies regarding the reproducibility of intraoral scanning (22 , 26). In addition, intraoral scanning was favored because it was used on dry skull dental surfaces, resulting in a lower number of errors compared to the clinical use, which is limited by patient restrictions (open-mouth limitation, absence of tongue movements, and saliva) (5). Qualitative results were represented by color dispersions after applying the deviation analyses, which referenced deviation values ranging from -0.3mm to 0.4mm. Color maps presented a threshold range of -1mm to 1mm to stipulate a control (semiautomatic superimposition) during the iteration of 3D meshes, similarly to findings in previous studies (22 , 25 , 27 , 28). However, the threshold (±1mm) was not described in earlier studies with similar purposes and methods to ours; it might have provided biased results regarding the error magnitude between superimposed 3D meshes (23 , 28). For both groups, the color maps were similar to each other. The darker tones of blue were observed on the occlusal sulci and/or fissures, and more orange and red tones on interproximal regions of the teeth, which most probably enabled a higher error magnitude (lower accuracy). In this sense, we may presuppose that, just as CBCT segmentations may interfere with 3D reconstructions, the interproximal regions and occlusal fissures/sulci of the teeth may present a higher predisposition towards impressions during the acquisition of models by optical scanning (26). In both groups, over 85% of the point combinations fall within the clinically acceptable deviation range. The deviation percentages of 0.5mm to -0.5mm were higher for the mandible superimpositions in IS (91.28%) and PM (91.1%). Comparatively, Nkenke et al. (8) showed that 44% of the mesh points were concentrated in deviations of 0.5mm for the maxilla, and 54% for the mandible. In the study by Gateno et al. (6), the average success rate for superimposing dental model file volumes with CBCT images was 72.3%, ranging from 59.8% to 80.2%. Although the methodologies used were not identical, due to the differences in CBCT and dental-model obtention techniques, our registration was apparently better than those in these studies (6 , 8). Comparing the first studies that used fiducial markers for superimposition processes between CBCT images and digital plaster models obtained with a laser scanner, the results showed a mean error value ranging from 0.1 to 0.5mm (6) and 0.1 to 0.3mm (2). In more recent studies, considering the similarity of the methodological aspects for superimpositions between CBCT and digital dental-model 3D meshes, the mean error values ranged from 0.27 to 0.33mm (27); mean deviation, from 0.11mm to 0.43mm, and RMS error from 0.13mm to 0.53mm (24); from 0.1mm to 0.28mm for the maxilla, and from 0.1mm to 0.34mm for the mandible (29); and from 0.32mm to 0.31mm for the maxilla and the mandible (28). When verifying the results of the Registration Error, MD+, and 3D Error, the values in Table 2 also approached the numerical values measured, falling within the variation range estimated by the studies described (24 , 27 - 29). Registration Error and 3D Error means and medians in the IS and PM group superimpositions of the maxillary arches presented higher values than in the mandibular arches, compatible with the previously described results where the maxilla superimpositions tend to have higher deviations than in the mandible (5 , 15). Notwithstanding, the comparisons between the IS and PM integrations for the maxilla did not show statistically significant differences for any of the variables. When analyzing the comparisons between the IS and PM registrations for mandibular arches, the results indicated the absence of statistically significant differences for the MD- and 3D Error variables. Nevertheless, Registration Error and MD+ values were lower in the IS group compared to the registration deviations (p&lt;0.05). To Swennen et al. (11), deviation magnitude related to the clinical significance would be unknown, but several studies stipulated a value of up to 0.5mm to refer to the clinically acceptable or irrelevant error magnitude in comparisons by linear measurements between plaster and digital models (28), or in the superimpositions of digital dental models over the 3D-mesh dental surfaces in CBCT images (6 , 8 , 24). Our results did not reject the hypothesis that the Registration Error, MD+, and 3D Error values were lower than or equal to 0.5mm, within the range established by Gateno et al. (3), Lin et al. (24), and Nkenke et al. (8). According to these studies, the 0.5mm borderline value could be related to Registration Error (25), deviation mean (MD+, for example) (26), and 3D Error or RMS (24). We agree that the 0.5mm value could be a borderline deviation for analyzing clinical viability during dental model superimpositions over CBCT images in virtual planning for orthognathic surgery and dental implants. Therefore, we have confidence in the substitutions of dental surfaces in the CBCT images. It's worth pointing out that we believe the RMS value (3D Error) was more determining as the error magnitude reference (accuracy), since it represents the degree of information shared in numerical values and the mutual reciprocity of combining points between the overimposed 3D meshes (24). Following the hypothesis proposal (H0: 0.5), it was also observed that none of the mean and median values were higher than or equal to 0.5mm in the 3D and vectoral deviations (Dx, Dy, and Dz), which confirmed the maintenance of a registration pattern between the 3D meshes, regardless of the overimposed regions. The 3D deviation values appeared to be influenced by the Dz vectoral deviations, as numerical-value proximity was observed between these deviations, possibly indicating that the error magnitudes were more significant in the superior-inferior direction. Nevertheless, there is a limitation in determining whether digital dental models tend to have a more superior or inferior deviated position (meshes with more spread-out surfaces), as all previous variable results were positive for the application of statistical tests and facilitated comparisons between the IS and PM groups. There is no comparison viability in the results from previous studies, even those with similar methodologies did not perform this type of measurement (8 , 24 , 27 - 29). There were no significant differences for most of the 3D and vectoral Dx, Dy, and Dz deviations. There was a significant statistical difference in the maxillary dental arch superimpositions, indicating a higher error magnitude in the deviation of 6 dental points for IS compared to PM. Whilst the mandibular dental arches also presented statistically significant 3D deviation values in 9 points, the magnitude of deviations for PM was higher than for IS. Nonetheless, these results represented a smaller deviation-difference proportion, as well as submillimetric values (m scale). The variability between them could also be related to the determination of the deviation points, which are arbitrated by an operator, potentially generating variations in the results due to human error. It´s also worth pointing out that the comparisons between the superimposition deviations on the anterior and posterior teeth did not represent determining differences for each group. In both groups, for both the maxillary and mandibular arches, there was a significant difference, with a higher proportion of deviations in the anterior teeth compared to the posterior teeth. Therefore, the posterior teeth regions did not seem to have an influence on the proposed methodology, unlike the findings of Grünheid et al. (30) and Sun et al. (22), which indicated greater deviations in the molar regions. The present study verified whether the magnitude of error would have the same performance pattern when integrating the different dental arches, regardless of the type of dental model. Although the error inherent in aligning different files, with limitations in model and image acquisition processing (especially CBCT), and human error bias, the results presented the same clinical application viability for both dental model types. Clinicians or software operators should recognize the acceptable error or deviation magnitude during CAD/CAM workflows that result from image file superimpositions, as it may directly impact the orientation coordinates used to determine dental implant placement or the position of the maxilla and mandible in orthognathic surgery. Even though guides tend to fit on the tooth surfaces, usually, significant differences may occur, and they may not achieve the proper positioning during the planned surgical procedure. The results showed a tendency to opt, whenever possible, for the intraoral scan, as it favored superimpositions over CBCT images, because the magnitude of the Registration Error was higher in the superimpositions with plaster models compared to those from the intraoral scanner. Nevertheless, there was no difference in 3D Error among the superimpositions with the different dental models, showing no detriment in the results obtained, regardless of the type of model being superimposed. It is essential to acknowledge, however, that this was an ex vivo study based on four dry skulls lacking soft tissues, which may not fully replicate clinical conditions. These aspects limit the generalizability of the findings and suggest caution when extrapolating the results to in vivo applications.

## Conclusions

The digital dental arch superimpositions over the CBCT images were not affected by the model-obtention method (mold or intraoral scan) for presenting the same performance, and both are acceptable for clinical applicability (deviations 0.5mm). Although the results did not indicate differences between the superimpositions of different dental models, the model superimpositions resulting from molding presented a greater magnitude of deviation (especially for the mandibular arches), which could suggest a tendency for the models obtained from the intraoral scan to favor superimpositions over those from CBCT images.

## Figures and Tables

**Table 1:** Table Descriptive results of the interaction-point percentage distributions according to the deviation range in which they were grouped.

Category	MX and MD					
	IS			PM		
	Mean (SD)	Median (IQR)	Min - Max	Mean (SD)	Median (IQR)	Min - Max
% clinically acceptable deviation	85.95 (±7.76)	88.63 (9.11)	66.68 - 95.72	85.20 (±7.18)	86.48 (10.15)	70.74 - 95.76
% clinically unacceptable deviation	11.96 (±6.05)	9.63 (7.05)	3.99 - 26.22	13.23 (±6.14)	12.33 (10.61)	4.18 - 24.02
Category	MX					
	IS			PM		
	Mean (SD)	Median (IQR)	Min - Max	Mean (SD)	Median (IQR)	Min - Max
% clinically acceptable deviation	80.80 (±7.51)	82.41 (9.5)	66.68 - 91.84	81.63 (±6.56)	82.61 (12.47)	70.74 - 90.99
% clinically unacceptable deviation	15.81 (±5.89)	14.90 (11.02)	7.56 - 26.22	15.94 (±5.13)	15.64 (10.11)	8.31 - 23.88
Category	MD					
	IS			PM		
	Mean (SD)	Median (IQR)	Min - Max	Mean (SD)	Median (IQR)	Min - Max
% clinically acceptable deviation	91.09 (±3.35)	91.28 (5.09)	85.72 - 95.72	88.78 (±5.94)	91.10 (9.36)	75.93 - 95.76
% clinically unacceptable deviation	8.1 (±2.99)	7.86 (4.22)	3.99 - 13.62	10.69 (±6.02)	7.92 (8.25)	4.18 - 24.02

IS: Intraoral device. MD: Mandible. MX: Maxilla. PM: Extraoral scanner. *p<0.05 for the Kruskal-Wallis and Mann-Whitney tests.

**Table 2:** Table Descriptive analysis results in mm.

Variables		MX and MD		MX	MD	MX	MD
		IS	PM	IS	IS	PM	PM
Registration Error	Mean (SD)	0.25 (±0.071)	0.27 (±0.06)	0.29 (±0.06)	0.20 (±0.03)	0.30 (±0.05)	0.24 (±0.06)
	Median (IQR)	0.23 (0.11)	0.27 (0.10)	0.3 (0.08)	0.19 (0.04)	0.29 (0.10)	0.22 (0.09)
	Min - Max	0.17 - 0.43	0.17 - 0.39	0.19 - 0.43	0.17 - 0.28	0.21 - 0.39	0.17 - 0.37
MD+	Mean (SD)	0.19 (±0.04)	0.21 (±0.06)	0.20 (±0.04)	0.18 (±0.04)	0.21 (±0.03)	0.22 (±0.08)
	Median (IQR)	0.17 (0.08)	0.20 (0.06)	0.20 (0.07)	0.16 (0.04)	0.21 (0.04)	0.18 (0.11)
	Min - Max	0.14 - 0.31	0.13 - 0.41	0.15 - 0.31	0.14 - 0.28	0.16 - 0.33	0.13 - 0.41
MD-	Mean (SD)	-0.31 (±0.09)	-0.31 (±0.07)	-0.36 (±0.09)	-0.25 (±0.04)	-0.35 (±0.07)	-0.27 (±0.05)
	Median (IQR)	-0.30 (0.12)	-0.31 (0.10)	-0.36 (0.15)	-0.25 (0.07)	-0.35 (0.10)	-0.26 (0.09)
	Min - Max	-0.55 - 0.20	-0.49 - 0.20	-0.55 - 0.23	-0.33 - 0.20	-0.49 - 0.24	-0.39 - 0.20
3D Error	Mean (SD)	0.36 (±0.09)	0.36 (±0.08)	0.4 (±0.08)	0.31 (±0.07)	0.39 (±0.06)	0.33 (±0.09)
	Median (IQR)	0.38 (0.15)	0.34 (0.13)	0.41 (0.13)	0.30 (0.12)	0.38 (0.11)	0.30 (0.10)
	Min - Max	0.22 - 0.53	0.22 - 0.55	0.26 - 0.53	0.22 - 0.45	0.29 - 0.53	0.22 - 0.55

IS: Intraoral device. MD: Mandible. MX: Maxilla. PM: Extraoral scanner.

## Data Availability

The datasets generated and analyzed during the current study are available from the corresponding author upon reasonable request.
